# Nd(III)-Induced Rice Mitochondrial Dysfunction Investigated by Spectroscopic and Microscopic Methods

**DOI:** 10.1007/s00232-015-9773-1

**Published:** 2015-02-04

**Authors:** Cai-Fen Xia, Long Lv, Xin-You Chen, Bo-Qiao Fu, Ke-Lin Lei, Cai-Qin Qin, Yi Liu

**Affiliations:** 1 School of Chemistry and Materials Science, Hubei Engineering University, Xiaogan, 432000 People’s Republic of China; 2 School of Chemistry and Food Sciences, Hubei University of Arts and Sciences, Xiangyang, 441053 People’s Republic of China; 3 State Key Laboratory of Virology & Key Laboratory of Analytical Chemistry for Biology and Medicine (MOE), College of Chemistry and Molecular Sciences, Wuhan University, Wuhan, 430072 People’s Republic of China

**Keywords:** Nd(III), Rice, Mitochondrial permeability transition (mPTP), Swelling, Transmembrane potential, Fluidity

## Abstract

The production capacity and yield of neodymium (Nd) in China have ranked the first in the world. Because of its unique biophysical and biochemical properties, Nd compounds have entered into the agricultural environment greatly to promote plant growth. Mitochondria play a crucial role in respiration and metabolism during the growth of plants. However, little is known about the mechanism by which Nd act at the mitochondrial level in plant cells. In this study, rice mitochondrial swelling, collapsed transmembrane potential and decreased membrane fluidity were examined to be important factors for mitochondria permeability transition pore (mPTP) opening induced by Nd(III). The protection of cyclosporin A (CsA) and dithiothreitol (DTT) could confirm that Nd(III) could trigger mPTP opening. Additionally, mitochondrial membrane breakdown observed by TEM and the release of cytochrome *c* (Cyt *c*) could also elucidate the mPTP opening from another point of view. At last, the study showed that Nd(III) could restrain the mitochondrial membrane lipid peroxide, so it might interact with anionic lipid too. This detection will be conductive to the safe application of Nd compounds in agriculture and food industry.

## Introduction

Rare earth elements (REEs) that possess unique physical and chemical properties are currently being extensively applied in medical, biochemical and agronomic fields. Lanthanum compounds have also been discovered that can stimulate the growth of agricultural products; hence, they have been widely used in farms as an important trace fertilizer (Guo et al. 1990). Cerium (Ce) is used an antiseptic agent for the treatment of burns (Monafo et al. 1976) in nanoparticle doping for hyperthermia (Kale et al. 2006) and in the large-scale affinity separation of proteins (Ma et al. 2005). As a result, the environment and food chain are being exposed to increasing numbers of REEs, which eventually accumulate in the human body. Other studies have showed the cytotoxicity of REEs, such as the capacity of Ce to cause the rice mitochondria dysfunction (Xia et al. 2013). Additionally, samarium (Sm) induced pathological changes, decreased the activity of superoxide dismutase (SOD) and increased malondialdehyde (MDA) concentrations in the liver and lung of rats (Shi et al. 2006).

At present, the production capacity and yield of neodymium (Nd) in China have ranked the first in the world (Wang et al. 2011). Because of its unique biophysical and biochemical properties, Nd compounds have entered into the agricultural environment greatly. In other words, the toxicity of Nd is being paid more and more attention in food nutrition and food safety. As reported, mitochondria play a crucial role in respiration and metabolism during the growth of plants (Logan 2006); however, little is known about the mechanism by which Nd act at the mitochondrial level in plant cells. Liu group had successfully verified that gadolinium (Gd), a heavy rare earth, could cause cell apoptosis by interacting with mitochondria (Zhao et al. 2014). In addition, it has been demonstrated the existence of the mitochondrial permeability transition pore (mPTP) in plants (Arpagaus et al. 2002).

In this background, we chose Nd(III), an important component of REE-based microfertilizers or additives, as a representative of light rare earth elements in next study and investigated the effect of Nd(III) on mitochondria especially for mPTP using isolated rice mitochondria as the model. By means of spectroscopy and microscopy, we analyzed the effects of Nd(III) on rice MPT through monitoring mitochondrial swelling, testing the transmembrane potential (Δ*Ψ*_m_), characterizing the fluidity of the membrane and observing mitochondrial ultrastructure in detail.

## Materials and Methods

### Chemicals

Cyclosporin A (CsA), rotenone, rhodamine 123 (Rh123), dithiothreitol (DTT), monobromobimane^+^ (MBM^+^), hematoporphrin (HP), oligomycin, valinomycin and MOPS were purchased from Sigma (St. Louis, MO). All other reagents were of analytical reagent grade, and all solutions were prepared with asepsis double-distilled water.

### Plant Material

Rice (Jiazao 935, China) seedlings were grown in water for about 10 days, in darkness, and at 30 °C. The water was changed twice daily. When the etiolated seedlings grew to 9–10 cm long, their stems (about 150 g fresh weight) were cut in small pieces and then used for isolation of mitochondria.

### Mitochondria Isolation

Rice mitochondria were isolated in accordance with standard differential centrifugation procedures as previously described (Vianello et al. 1995), with minor modifications. The extraction medium contained 0.4 M sucrose, 1 mM EDTA, 30 mM Tris–HCl, 4 mM cysteine, 0.6 % PVP and 0.1 % BSA, and the pH was adjusted to 7.5. The wash medium contained 0.3 M sucrose, 1 mM EDTA, and 10 mM Tris–HCl and was regulated to pH 7.5. Rice stems were peeled, then transferred to 250 mL of extraction medium, and disrupted in a waring blender at intermediate speed for 4 × 10 s, then at high speed for 4 × 10 s. The filtrate was then centrifuged at 1,500×*g* for 10 min and the pellet was discarded. The mitochondria were collected by centrifugation at 11,000×*g* for 8 min, resuspended in about 10 mL wash medium. So thick mitochondria were gained. For purification of rice mitochondria, portions (1 mL) of the final pellet were layered on top of gradients consisting of 50 mL of buffer containing 0.3 M sucrose, 0.3 M mannitol and 50 mM Tris–HCl. The mixture was centrifuged at 11,000×*g* for 6 min. The purification of mitochondria was operated again, except the volume was changed to 30 mL. Mitochondrial protein was determined by Bradford method using BSA as a standard.

### Swelling Measurement

Mitochondrial osmotic volume changes were measured spectrophotometrically by monitoring the absorbance at 540 nm over 10 min at 25 °C (Pastore et al. 1999). Mitochondria (0.4 mg/mL protein) were suspended in 2 mL respiration buffer (250 mM sucrose, 20 mM HEPES, 2 mM MgCl_2_, 5 mM KH_2_PO_4_, 20 mM succinate and 1 µM rotenone, pH 7.2) and incubated with different concentrations of Nd(Ш). Spectra were recorded on a MAPADA UV-61 100PC double beam spectrophotometer equipped with 1.0-cm quartz cells.

Mitochondrial inner membrane permeabilization to K^+^ and H^+^ in the presence of Nd(Ш) was detected in potassium nitrate medium and potassium acetate medium, respectively (Fernandes et al. 2006). The K-nitrate medium contained 13.5 mM KNO_3_, 5 mM HEPES (pH 7.1), 0.1 mM EGTA, 0.2 mM EDTA and 2 µM rotenone, while the K-acetate medium contained 135 mM K-acetate, 5 mM HEPES (pH 7.1), 0.1 mM EGTA, 0.2 mM EDTA, 1 µg/mL valinomycin and 2 µM rotenone.

Meanwhile, in order to understand the interaction site or sites of high concentrations of Nd(Ш) on rice mitochondria, the effects of DTT, CsA, MBM^+^, ADP and EDTA on mitochondrial swelling were also detected (Liu et al. 2011).

### Transmembrane Potential Measurement

The membrane potential was measured indirectly using Rhodamine 123 as an optical probe (Fu et al. 2006). The fluorescence intensity change of Rh123 was recorded, thermostated at 30 °C by a luminescence spectrometer, model LS 55. The excitation and emission wavelengths were 488 and 530 nm, respectively. A slit width of 10 nm for both emission and excitation was set. The calibration of Rh123 intensity changes, as a function of Nd(Ш) diffusion potentials, was basically performed. Rice mitochondria (0.4 mg/mL protein) were firstly incubated for 10 min in 2.5 mL of the respiratory medium, then labeling with 100 nM Rhodamine 123 for 5 min.

### Membrane Fluidity Assessment

Fluidity changes of the mitochondrial membranes were evaluated by the changes of fluorescence excitation anisotropy (*r*) of mitochondria-bound dyes (Ricchelli et al. 1999). In our experiments, hematoporphrin IX (HP) was used. Stock solutions of the probe were prepared in absolute ethanol. HP (final concentration 6 µM) was injected into the rice mitochondrial suspensions (0.4 mg/mL protein) and incubated for 5 min before monitoring anisotropy. Anisotropic changes were recorded by the LS 55 luminoscope at *λ*_ex_ = 520 nm, *λ*_em_ = 626 nm for HP. The fluorescence anisotropy *r* is defined by the equation:

$$r = {{\left( {I_{\text{VV}} - {\text{GI}}_{\text{VH}} } \right)} \mathord{\left/ {\vphantom {{\left( {I_{\text{VV}} - {\text{GI}}_{\text{VH}} } \right)} {\left( {I_{\text{VV}} + 2{\text{GI}}_{\text{VH}} } \right)}}} \right. \kern-0pt} {\left( {I_{\text{VV}} + 2{\text{GI}}_{\text{VH}} } \right)}}$$

where *I*_VV_ and *I*_VH_ are the fluorescence intensities polarized parallel and perpendicular to the vertical plane of polarization of the excitation beam, respectively, and *G* is the correction factor for instrumental artifacts. All the assays were performed at 25 °C.

### TEM of Mitochondria

Control and neodymium-treated mitochondria were collected and fixed in 2.5 % (v/v) glutaraldehyde in 50 mM cacodylate buffer, pH 6.8, for 1 h at 4 °C, then postfix with 2 % osmium tetroxide in the same buffer for 1 h at room temperature and dehydrated (Gzyl et al. 2009). The ultrastructure of mitochondria was observed with a JEM-100CX transmission electron microscope.

### Measurement of *Cyt*-*c* Release by ELISA

Rice mitochondria were suspended in respiration buffer and incubated with different concentrations of Nd(III) at 30 °C for 30 min. The suspension was then centrifuged for 15 min at 11,000×*g*. The supernatants were analyzed by *Cyt*-*c* assays (all in triplicate) using ELISA kit (Yuan et al. 2003). The 96-well polyvinyl preparation plates were coated with anti-*Cyt*-*c* polyclonal antibody and then blocked with 0.5 % gelatin in PBS for 1 h. Into the wells, 100 μL of samples or standards were added and incubated at 37 °C for 40 min. Cyt-c antibody was added to the wells and incubated at 37 °C for 40 min. Then HRP-antibody conjugate was added and incubated at 37 °C for 40 min. The wells were washed three times with washing buffer before each step above. Finally, 100 μL of substrate and 50 μL of stop solution were added sequentially. The absorption at 450 nm was recorded on microplate reader (Biotek, Elx 800). The release amount of Cyt *c* was calculated according to the standard curve equation.

## Results

### Nd(III)-Induced Rice Mitochondrial Swelling

**Fig. 1:**
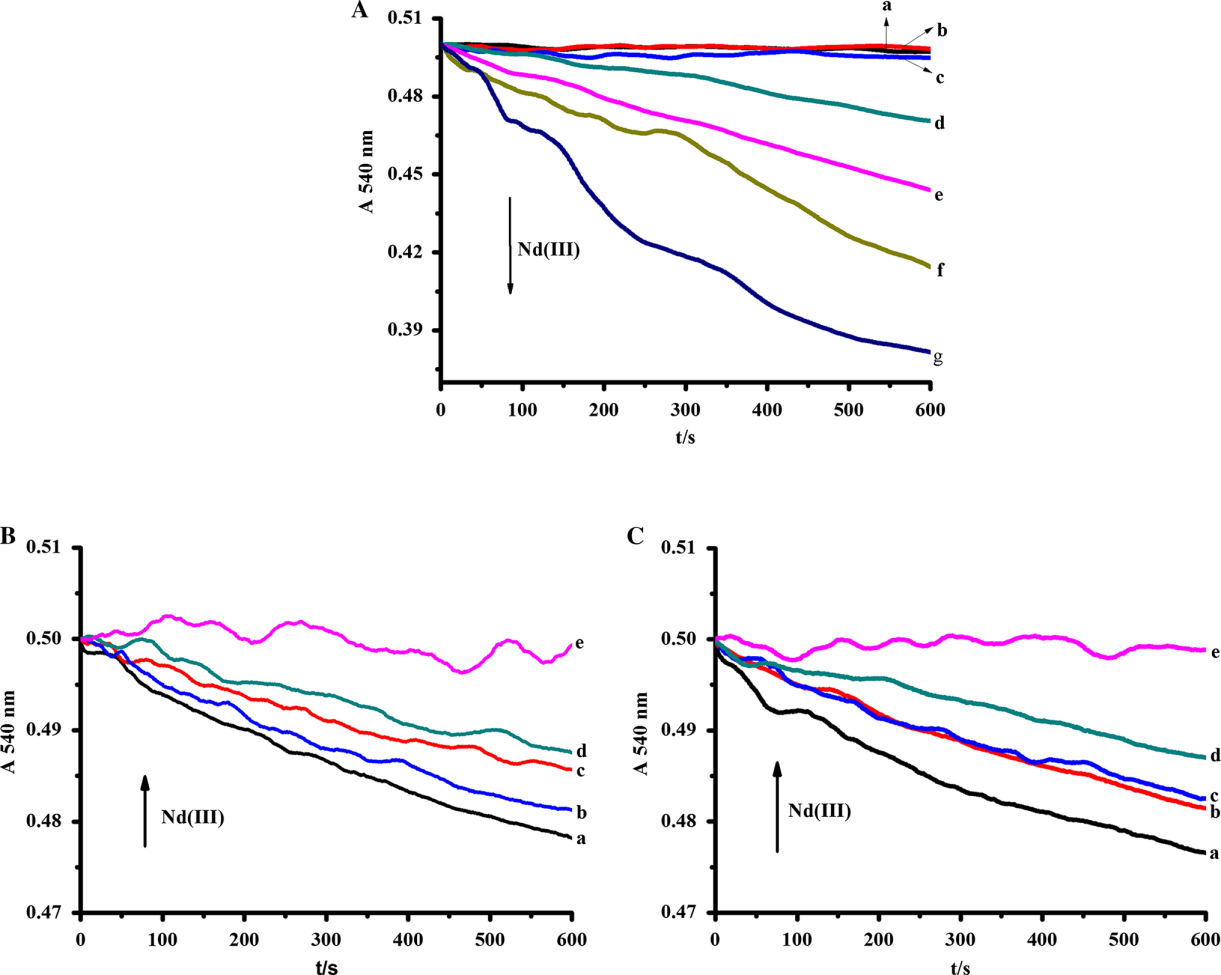
**a** Swelling of isolated rat liver mitochondria caused by different concentrations of Nd(III). *c*(Nd^III^)/μM (*a*–*g*): 0, 50,100, 200, 300, 400, 500. **b** Effect of different concentrations of Nd(III) on mitochondrial inner membrane permeabilization to K^+^. *c*(Nd^III^)/μM (*a*–*e*): 0, 50, 100, 200, 400. **c** Effect of different concentrations of Nd(III) on mitochondrial inner membrane permeabilization to H^+^. *c*(Nd^III^)/μM (*a*–*e*): 0, 50, 100, 200, 400

The effects of different concentrations of Nd(Ш) on mitochondrial swelling were evaluated in the light of decrease in absorbance at 540 nm (*A*_540_) over 10 min. The results were displayed in Fig. 1a. Low concentrations of Nd(Ш) (0–100 µM) hardly triggered mitochondrial swelling, but Nd(Ш) induced mitochondrial swelling at high concentrations (200–500 µM) with a proportional dependence of the swelling tendency on the concentration of Nd(Ш). It was obvious that Nd(Ш) at higher concentration was more effective than it at lower concentration on rice mitochondrial osmotic volume changes.

As shown in Fig. 1b, rice mitochondria undergo a rapid swelling rate in K-nitrate solution without Nd(III). Unlike the fact, the curves indicated that mitochondrial matrix was condensed in a concentration-dependent manner in the presence of Nd(III). Similarly, mitochondrial inner membrane permeabilization to H^+^ was also reduced along with increasing concentration of Nd(III) in Fig. 1c.

### Effects of Nd(Ш) on Mitochondrial Transmembrane Potential

**Fig. 2:**
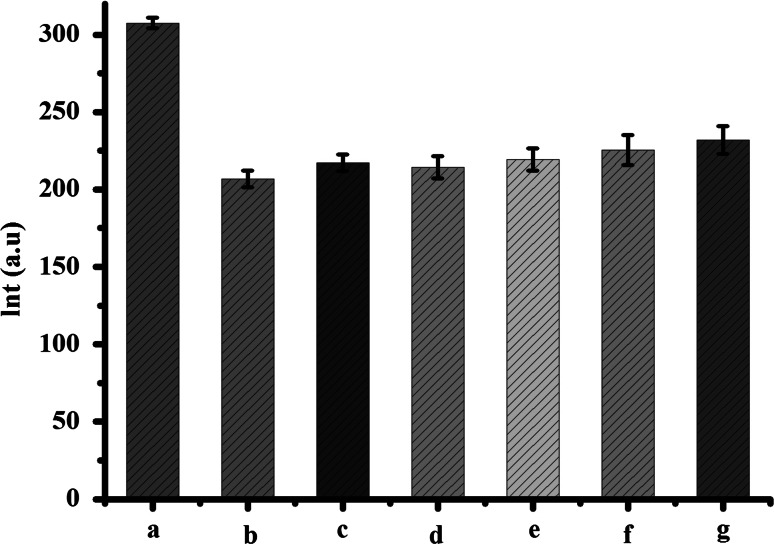
Mitochondrial membrane potential (Δ*Ψ*_m_) measurement, which was followed with the indicator Rh123. Column *a*–*f* represented the addition of Nd(III), *c*(Nd^III^)/μM: 0, 50, 100, 200, 300, 400. Column *g* was only 100 nM Rh123

Mitochondrial transmembrane potential (∆*Ψ*_m_) was monitored using Rh123 staining. Rh123 accumulates in the rice mitochondrial matrix due to the inside negative ∆*Ψ* of the mitochondrial inner membrane, leading to fluorescence quenching of Rh123 (Column a in Fig. 2). In the case of membrane depolarization, Rh123 is released into the medium, causing increase in its fluorescence intensity. As shown in Fig. 2(Column b–f), incubation of mitochondria with Nd(Ш) increased the fluorescence intensity of the Rh123 labels. Because low ∆*Ψ*_m_ corresponds to high value of fluorescence in these tests (Zhu et al. 2002), the results indicated that ∆*Ψ*_m_ was impaired by the addition of Nd(Ш). Interestingly, at low concentrations (0–200 µM) of Nd(Ш), ∆*Ψ*_m_ was a little collapsed; and at higher concentrations (300–400 µM), ∆*Ψ*_m_ was dissipated obviously.

### Effects of Nd(Ш) on Mitochondrial Membrane Fluidity

**Fig. 3:**
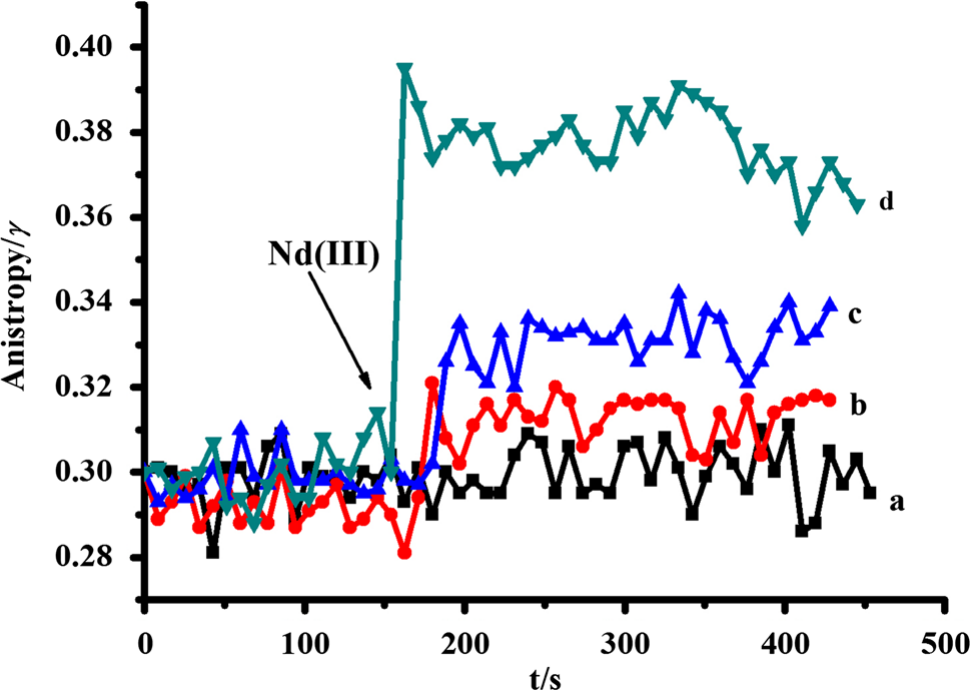
Changes in mitochondrial membrane fluidity caused by different concentrations of Nd(Ш). Trace *a*–*d*, *c*(Nd^III^)/μM: 0, 100, 300, 500

Membrane permeability transition of mitochondria was often accompanied with by fluidity changes of its membranes. HP mostly interacted with biological membranes protein sites and was selectively located on the mPTP constituents (Dong et al. 2013). The change of anisotropy reflected membrane fluidity change and mPTP conformational variation (Ricchelli et al. 1999). In order to detect whether the effects of Nd(Ш) were related to rice mitochondrial membrane fluidity, the changes in the steady-state anisotropy of mitochondria-bound HP were analyzed by spectroscopic methods. The results are shown in Fig. 3. The HP anisotropy was continuously monitored, and a gradual increase in the anisotropy of HP (from 0.30 to 0.40) was observed with the addition of different concentrations of Nd(Ш). Furthermore, the increase in HP anisotropy was dose dependent in the experimental concentrations. Since the decrease in HP anisotropy corresponded to the increase in membrane fluidity, the rigidness of isolated rice mitochondrial membrane was enhanced in the presence of Nd(Ш) from the graph.

### Effects of Nd(Ш) on Mitochondrial Ultrastructure

**Fig. 4:**
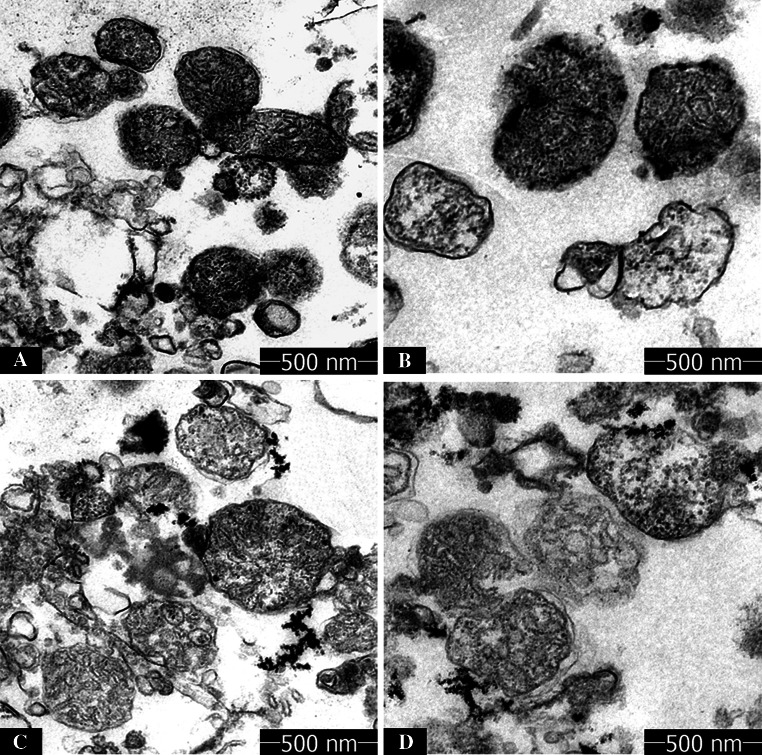
Effects of Nd(III) on rice mitochondrial ultrastructure mitochondria were incubated without (**a**) or with Nd(Ш) at 100 (**b**), 300 (**c**), 500 (**d**) µM

To better understand the mitochondrial permeability transition induced by Nd(Ш), the ultrastructure of rice mitochondria treated with or without Nd(Ш) was investigated by TEM. Mitochondria extracted from rice root maintained their integrity with the classical ultrastructure, which embodied a well-defined outer membrane, a narrow intermembrane space and compact cristae as shown in Fig. 4a. Mitochondrial shape has no obvious change, demonstrating that low concentrations of Nd(III) affect mitochondria in a low degree (Fig. 4b). Inversely, presence of 500 µM Nd(Ш) caused mitochondrial structure broken down, and mitochondria appeared a spherical configuration with reduced matrix electron density and a large intermembrane space (Fig. 4d).

Based on the results stated above, we draw a conclusion to a great extent that rice MPT was hardly induced by low concentrations of Nd(Ш), while at high concentrations, the addition of Nd(Ш) apparently triggered mPTP opening. To clarify the mechanism of these effects, further tests were conducted necessarily.

### Possible Mechanism of High Dose of Nd(Ш) on Rice Mitochondria

**Fig. 5:**
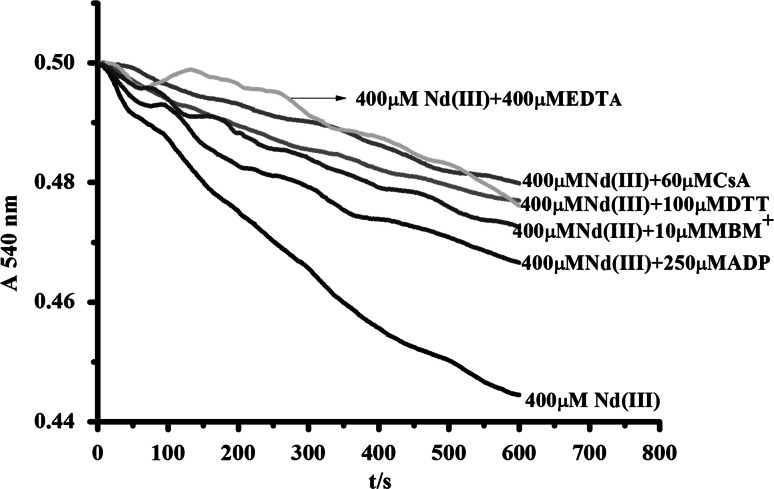
Inhibition of CsA, DTT, MBM + , ADP, EDTA on MPT induced by 400 μM Nd(III)

DTT is a disulfide reductant and could restrain the mPTP opening caused by oxidation of -SH on membrane protein of mitochondria (Zoratti and Szabò 1995). MBM and its cationic derivative MBM^+^ could prevent mPTP opening by reacting with some dithiol oxidants or cross-linkers (Zhang et al. 2011). CsA is considered as a classical inhibitor of the mPTP opening and used as the identifying characteristic of the mPTP opening (Zoratti et al. 2005). CsA inhibited mPTP opening through interaction with protein CyP-D between outer and inner membrane. ADP could modulate mPTP opening by controlling ANT conformation. As shown in Fig. 5, DTT, MBM^+^, CsA and ADP could suppress mitochondrial swelling induced by 400 μM Nd(III). Moreover, EDTA also inhibited mitochondrial swelling after removal of Nd(III) by chelation.

### Effect of Nd(III) on Mitochondrial Membrane Lipid Peroxidation

**Fig. 6:**
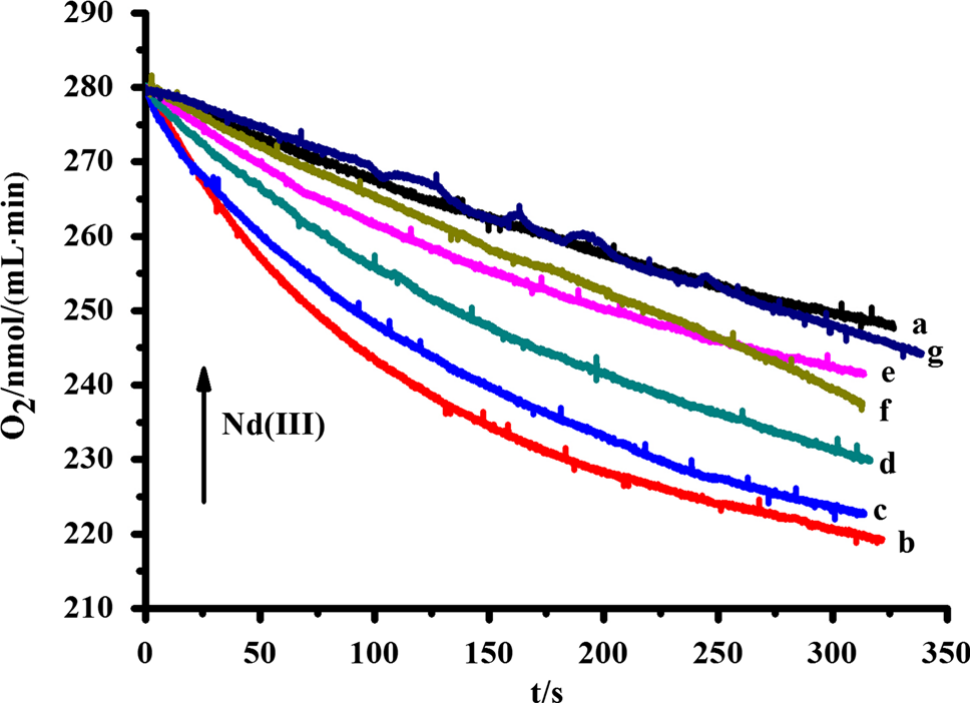
Effect of Nd(III)on membrane lipid peroxidation of rice mitochondria induced by the pro-oxidant pair ADP/Fe^2+^. Traces *a* represented control without ADP/Fe^2+^, traces *b*–*g* represented the addition of Nd(III) with ADP/Fe^2+^, *c*(Nd^III^)/μM: 0 (*b*),100 (*c*), 200 (*d*), 300 (*e*), 400 (*f*), 500 (*g*)

Membrane lipid peroxidation induced by the pro-oxidant pair ADP/Fe^2+^ involved two phase kinetics in oxygen consumption: an initial lag phase and the rapid oxygen consumption phase. The lag phase was probably related to the time required for the generation of adequate ion complex (ADP − Fe^2+^ − O_2_ ↔ ADP − Fe^3+^ − O_2_^−^) which is responsible for the initiation of lipid peroxidation (Fernandes et al. 2006). The rapid oxygen consumption phase was probably due to the oxidation of the polyunsaturated fatty acid acyl chain on membrane phospholipids by reactive oxygen species (Niki et al. 2005). As shown in Fig. 6, Nd(III) reduced rapid oxygen consumption in a dose-dependent manner, indicating that Nd(III) inhibited the initiation of mitochondrial membrane lipid peroxidation.

### Effect of Nd(III) on the Release of Cyt *c*

Cyt *c* is associated with the inner membrane through electrostatic interaction with acidic phospholipids, complex III and IV, as well as the hinge protein (Taresa et al. 1992; Jutila et al. 1998; Subramanian et al. 1998). Cyt-*c* release is influenced by the integrity of the outer mitochondrial membrane, so the opening of mPTP caused mitochondrial membrane damage and the release of Cyt *c*, which ultimately resulted in apoptosis (Nageswara and Marschall 2007). Table 1 shows that Cyt *c* was released from isolated mitochondria incubated with Nd(III) during the incubation period.

**Table 1:** Effect of Nd(III) on release of Cyt *c* from rice mitochondria

*c*(Nd^III^)/μM	OD(450 nm)	*c*(Cyt c)/nM
0	0.298	24
100	0.297	24
300	0.339	30
500	0.313	26

## Discussion

Lanthanides have been shown to be able to induce several types of cell apoptosis (Liu et al. 2003). Similarly, Nd(III) could combine with cellular membranes, which may interfere with mitochondrial normal function. Mitochondria play a crucial role in regulation of cell apoptosis, which can be preceded by the occurrence of mPTP. Our work centralized on the effect of Nd(III) on mPTP. The study demonstrated that high dose of Nd(III) (200–500 μM) caused rice mitochondrial swelling, dissipation of ∆*Ψ*_m_ and decreased membrane fluidity.

Mitochondrial swelling is believed to be one of the most important indicators of the opening of the mPTP and a sign of mitochondrial dysfunction (Tang et al. 2005; Gerencser et al. 2008). Swelling is commonly monitored as a fast decrease in the absorbance of the mitochondrial suspension (Passarella et al. 2003). Consistently, the experimental shown in Fig. 1 appeared to agree well with those reports. The high concentrations of Nd(Ш) (200–500 μM) could lead to the occurrence of swelling, which indicated mitochondrial outer membrane permeabilization function was disturbed and mitochondrial matrix concentration changes might be caused by mPTP opening. In most cases, mPTP opening was accompanied by the change of membrane fluidity related to the increase in membrane permeability (Rolo et al. 2003). As shown in Fig. 3, the membrane fluidity decreased and probed by HP, indicating the inner membrane protein regions involved mPTP formation, was changed (Malekova et al. 2007). Both mitochondrial transmembrane potential and Cyt c were also important in keeping the regular function of mPTP (Kulikov et al. 2012). High dose of Nd(III) induced dissipation of ∆*Ψ*_m_ and the release of Cyt *c,* respectively, demonstrating mPTP opening again.

Mitochondrial inner membrane is only freely permeable to oxygen, water and some proteins. Acetate anion and H^+^ go through the mitochondrial inner membrane, then dissociate in the matrix, producing a proton gradient (Liu et al. 2011). As shown in Fig. 1b, Nd(III) could destroy this proton gradient to suppress the opening of inner membrane permeability transition pore, so that the process of oxidative phosphorylation might be influenced consequently.

On one hand, the protective effect of DTT and CsA confirmed that mitochondrial swelling primarily occurred as a result of mPTP opening. On the other hand, TEM indicated that high concentration of Nd(III) caused rice mitochondrial swelling and destroyed inner membrane integrity. EDTA cannot enter matrix because it is impermeable to the membrane. Therefore, Nd(III) induced rice mPTP but did not need to enter the mitochondrial matrix. As reported, the S site, a critical dithiol, has been proposed to contribute to MPT modulation (Bernadi 1996). In our study, MBM^+^ could partly reverse the swelling due to its protection on “S” on the inner membrane external face (Leung and Halestrap 2008). Moreover, the protective effect of DTT indicated that mitochondrial swelling caused by high concentration of Nd(III) was associated with -SH groups on internal face of the mitochondrial inner membrane. Accordingly, the findings could prove that Nd(III) caused rice mitochondria dysfunction via bindings “S” site on inner membrane.

It is worth noting that Nd(III) reduced the rapid oxygen consumption of mitochondrial lipid peroxidation. In other words, ROS was not the main factor during the process of mitochondria oxidative phosphorylation and Nd(III) only interacted with anionic lipids. So the results demonstrated that Nd(III) exerted its effect on mPTP opening by interacting with multiple sites not exclusively proteins but by anionic lipids as well.

## Conclusion

In this study, the rice mitochondrial dysfunction with the addition of Nd(III) was investigated by spectroscopic and microscopic methods. Nd(III) had little influence on mitochondria at low concentrations (< 200 μM). But Nd(III) could induce swelling, collapse membrane potential, decrease membrane fluidity and change mitochondrial ultrastructure, which indicated that Nd(III) caused mPTP opening at high concentrations (200–500 μM). A series of protective experiment proved that Nd(III) caused rice mitochondria dysfunction by interacting with multiple proteins on outer and inner membrane. Notably, Nd(III) had influence on membrane lipid peroxidation in rapid oxygen consumption phase. Hence, Nd(III) was supposed to influence mPTP opening not only through interacting with proteins but also with anionic lipids.
